# Acknowledgment to reviewers of *Extracellular Vesicles and Circulating Nucleic Acids* in 2024

**DOI:** 10.20517/evcna.2025.05

**Published:** 2025-02-08

**Authors:** EVCNA Editorial Office

**Affiliations:** Suite 1504, Tower A, Xi’an National Digital Publishing Base, No. 996 Tiangu 7th Road, Gaoxin District, Xi’an 710077, Shaanxi, China.

In 2024, *EVCNA* reached remarkable milestones, achieving indexing in two prestigious international databases: ESCI (Web of Science) and PMC. These accomplishments would not have been possible without the unwavering support of researchers devoted to the growth and success of *EVCNA*. The *EVCNA* Editorial Office extends its heartfelt gratitude and sincere appreciation to the reviewers [Table 1] who generously dedicated their time and expertise to evaluating manuscripts throughout the year. Your invaluable contributions, regardless of whether the reviewed papers were ultimately published, have been instrumental in upholding the quality and integrity of our journal. Thank you for your exceptional commitment and efforts!

**Table 1:** Reviewers in 2024

Zhang, Daniel Xin (United States)	Sgambato, Alessandro (Italy)	Müller, Janis A (Germany)
Fontana, Simona (Italy)	Franquesa, Marcella (Spain)	Schaack, Beatrice (France)
Catalano, Myriam (Italy)	CABAÑAS, Carlos (Spain)	Ji, Peifeng (China)
Bemis, Lynne (United States)	Chutipongtanate, Somchai (South Korea)	Kaur, Sukhbir (United States)
Chandler, Natalie (United Kingdom)	Gupta, Dhanu (Sweden)	Martín-Cófreces, Noa Beatriz (Spain)
Celec, Peter (Slovakia)	Kolesar, Jill Marie (United States)	Jeffrey, Franklin (United States)
Campbell, Phil G (United States)	Kanada, Masamitsu (United States)	Falasca, Marco (Italy)
Das, Saumya (United States)	Altosaar, Illimar (Canada)	Kuehn, Meta (United States)
Lorico, Aurelio (United States)	Grégory, Christopher D. (United Kingdom)	Liguori, Giovanna L. (Italy)
Lugini, Luana (Italy)	Poggi, Alessandro (Italy)	Gonzalez-Kozlova, Edgar E. (United States)
Vaquero, Javier (Spain)	Yang, Yoosoo (South Korea)	Denis, Gerald V. (United States)
Tatischeff, Irène (France)	Malagelada, Cristina (Spain)	Crescitelli, Rossella (Sweden)
Zubair, Abba Chedi (United States)	Lee, Bao-Hong (Taiwan, China)	Russo, Domenico (Italy)
Saha, Banishree (United States)	Jay, Steven M. (United States)	Leor, Jonathan (Israel)
Kong, Jiming (Canada)	Katakura, Yoshio (Japan)	Suades, Rosa (Spain)
Carata, Elisabetta (Italy)	Brucale, Marco (Italy)	COMINCINI, SERGIO (Italy)
Engelbrecht, Anna-Mart (South Africa)	Guan, Xingang (China)	Rodosthenous, Rodosthenis S. (Finland)
Toh, Wei Seong (Singapore)	Peake, Nicholas J. (United Kingdom)	Sourisseau, Marion (United States)
Bazzan, Erica (Italy)	Morelli, Adrián E. (United States)	Pegtel, Dirk (Netherlands)
Silva, Saimon (Australia)	Brambilla, Marta (Italy)	Giannecchini, simone (Italy)
Lettau, Marcus (Germany)	Ma, Zhaowu (China)	Lundy, David J (Taiwan, China)
Brizzi, Maria Felice (Italy)	Kwan, Hiu Yee Anna (Hong Kong, China)	Ahn, Miwon (United States)
Blanco-López, M. C. (Spain)	Buchet, Rene (France)	Ghirardello, Anna (Italy)
Luwor, Rodney (Australia)	Bottini, Massimo (Italy)	Janbon, Guilhem (France)
Badiavas, Evangelos V. (United States)	Navas-Martin, Sonia (United States)	Romani, Rita (Italy)
Iaccino, Enrico (Italy)	Ruan, Shaobo (China)	Jung, Anna Lena (Germany)
Koning, Roman (Netherlands)	Miron, Richard J. (Switzerland)	Greening, David W. (Australia)
Dávalos, Alberto (Spain)	Chen, Guang (China)	Amadio, Marialaura (Italy)
Zempleni, Janos (United States)	Driss, Adel (United States)	Ageta, Hiroshi (Japan)
Borrás, Consuelo (Spain)	Faria Pais, Teresa (Portugal)	Sorice, Maurizio (Italy)
Ghatak, Subhadip (United States)	Gazarini, Marcos Leoni (Brazil)	Rizvanov, Albert Anatolyevich (Russian Federation)
Russell, Ashley E. (United States)	Roca, Jordi (Spain)	Luo, Weiren (China)
Da Silveira, Juliano Coelho (Brazil)	Reiners, Katrin S. (Australia)	Weidenhofer, Judith (Australia)
Gabrielsson, Susanne (Sweden)	Lucien, Fabrice (United States)	Chiabotto, Giulia (Italy)
De la Cuesta, Fernando (Spain)	Potaczek, Daniel P. (Germany)	Bruno, Stefania (Italy)
Iwata, Hisataka (Japan)	Melo, Sónia A. (Portugal)	Heuchel, Rainer (Sweden)
Zhao, Xiangxuan (China)	Tryggestad, Jeanie B. (United States)	Brady, Jeannine L (United States)
Dini, Luciana (Italy)	Liu, Han (China)	D'Agostino, Vito G. (Italy)
Situ, Bo (China)	Prados-Rosales, Rafael C. (Spain)	Urbini, Milena (Italy)
Vago, Riccardo (Italy)	Badía, Josefa (Spain)	Richards, Christopher I. (United States)
Shiba, Kiyotaka (Japan)	Vinokurova, Svetlana V. (Russian Federation)	Chi, Zailong (China)
Meenakshisundaram, Gopinath (Singapore)	Ding, Xianguang (China)	Petit, Isabelle (France)
Chaiswing, Luksana (United States)	Foschino Barbaro, Maria Pia (Italy)	Yamamoto, Yusuke (Japan)
Zhao, Libo (China)	Bart, Geneviève (United Kingdom)	Zhang, Xudong (China)
Dutta, Suman (United Kingdom)	Behringer, Erik (United States)	Ferrero, Hortensia (Spain)
Park, Ki Soo (South Korea)	Smith, Roger (Australia)	Roura, Santiago (Spain)
Salzig, Denise (Germany)	Shen, Tang-Long (Taiwan, China)	Neri, Tommaso (Italy)
Jaimovich, Enrique (Chile)	Whiteside, Theresa L. (United States)	Chrzanowski, Wojciech (Australia)
Morasso, Carlo Francesco (Italy)	Graner, Michael (United States)	Lingg, Nico (Austria)
Wang, Chao (China)	Huang, Shenglin (China)	Yoon, Daesung (South Korea)
Kou, Xiaoxing (China)	Nakayasu, Ernesto Satoshi (United States)	Rinner, Beate (Austria)
Urbanelli, Lorena (Italy)	Huang, Zhaohui (China)	Brachner, Andreas (Austria)
Chen, Yong (China)	Turko, Illarion V. (United States)	Jiang, Cheng (China)
Yoshioka, Yusuke (Japan)	Deng, Zaian (China)	Ferraro, Davide (Italy)
Royo, Luis J (Spain)	Qadri, Shahnaz M. (United States)	Wang, Yansong (China)
Kaeffer, Bertrand B. (France)	Ribeiro-Rodrigues, Teresa M. (Portugal)	Laumet, Geoffroy (United States)
Garofalo, Tina (Italy)	Patton, James (United States)	Zhong, Tianyu (China)
Beck, Hans Christian (Denmark)	Droho, Steven (United States)	Seufferlein, Thomas Theodor Werner (Germany)
Porter, Ryan (United States)	Luo, Zhiwen (China)	Cooks, Tomer (Israel)
Rai, Alex J. (United States)	Ross, Kehinde (United Kingdom)	Lewis, Stephen M. (Canada)
Gallart-Palau, Xavier (Spain)	Mukhopadhyay, Arijit (United Kingdom)	Han, Guanghong (China)
Ochieng, Josiah (United States)	Haqqani, Arsalan (Canada)	Taverna, Simona (Italy)
Yu, Fabiao (China)	Bravo Lopez, Susana Belén (Spain)	Han, Pingping (Australia)
López de las Hazas, María Carmen (Spain)	Altei, Wanessa (Brazil)	Bilousova, Tina (United States)
Ribitsch, Iris (Austria)	Preußer, Christian (Germany)	Ménasché, Philippe (France)
Zheng, Lei (China)	Breyne, Koen (United States)	Berezin, Alexander E. (Austria)
Zhang, Ye (China)	Borthakur, Alip (United States)	Menck, Kerstin (Germany)
Garcia-Silva, Susana (Spain)	Yáñez-Mó, María (Spain)	Lei, Jianyong (China)
Teixeira, Ana Luísa (Portugal)	Webber, Jason (United Kingdom)	Liu, Hengrui (United Kingdom)
Luo, Lan (China)	Kodali, Maheedhar (United States)	Neuhaus, Winfried (Austria)
Luirink, Joen (Netherlands)	Yuyama, Kohei (Japan)	Seo, Naohiro (Japan)
Sui, Bingdong (China)		

The invaluable contributions of our expert reviewers are central to the journal’s editorial decision-making process. As a gesture of gratitude, we established individual reviewer accounts in the submission system in 2024. These accounts enable reviewers to access details of their past reviews, view comments from fellow reviewers, and download letters of acknowledgment for their records.

To further express our appreciation, the Editorial Office has selected two exceptional individuals as the “2024 Best Reviewers.” Each awardee will receive a prize of USD 350, with the winners to be announced in March 2025.

Once again, we extend our heartfelt thanks to all our reviewers for their generosity and dedication throughout the past year. We look forward to continuing our collaboration in 2025 to uphold a rigorous peer review process and maintain the high publishing standards of *EVCNA*!

## DECLARATIONS

### Copyright

©The Author(s) 2025.

